# The Three-Dimensional In Vitro Cell Culture Models in the Study of Oral Cancer Immune Microenvironment

**DOI:** 10.3390/cancers15174266

**Published:** 2023-08-25

**Authors:** Elaheh Dalir Abdolahinia, Xiaozhe Han

**Affiliations:** Department of Oral Science and Translation Research, College of Dental Medicine, Nova Southeastern University, Fort Lauderdale, FL 33314, USA

**Keywords:** oral cancer, extracellular matrix, two/three-dimensional (2D/3D) model, immune cells, tumor cells, stromal cell

## Abstract

**Simple Summary:**

The extensive array of applications in 3D culture models closely mimics the precise physiological conditions of in vivo settings, enabling the investigation of anticancer drug resistance, which suggests that, in addition to genes, cell structure significantly impacts toxicity outcomes. Commercial 3D culture systems offer a valuable platform to explore various facets of oral cancer research. These models present an ethical and reproducible alternative to animal studies, facilitating the examination of oral microbial population dynamics, cellular interactions between cancer cells and immune cells, and the role of macrophage cells in oral cancer.

**Abstract:**

The onset and progression of oral cancer are accompanied by a dynamic interaction with the host immune system, and the immune cells within the tumor microenvironment play a pivotal role in the development of the tumor. By exploring the cellular immunity of oral cancer, we can gain insight into the contribution of both tumor cells and immune cells to tumorigenesis. This understanding is crucial for developing effective immunotherapeutic strategies to combat oral cancer. Studies of cancer immunology present unique challenges in terms of modeling due to the extraordinary complexity of the immune system. With its multitude of cellular components, each with distinct subtypes and various activation states, the immune system interacts with cancer cells and other components of the tumor, ultimately shaping the course of the disease. Conventional two-dimensional (2D) culture methods fall short of capturing these intricate cellular interactions. Mouse models enable us to learn about tumor biology in complicated and dynamic physiological systems but have limitations as the murine immune system differs significantly from that of humans. In light of these challenges, three-dimensional (3D) culture systems offer an alternative approach to studying cancer immunology and filling the existing gaps in available models. These 3D culture models provide a means to investigate complex cellular interactions that are difficult to replicate in 2D cultures. The direct study of the interaction between immune cells and cancer cells of human origin offers a more relevant and representative platform compared to mouse models, enabling advancements in our understanding of cancer immunology. This review explores commonly used 3D culture models and highlights their significant contributions to expanding our knowledge of cancer immunology. By harnessing the power of 3D culture systems, we can unlock new insights that pave the way for improved strategies in the battle against oral cancer.

## 1. Introduction

Head and neck cancers (HNCs), positioned as the seventh most prevalent cancer on a global scale, carry a significant burden of mortality. In 2018 alone, staggeringly, more than 170,000 deaths were attributed to HNCs, which also exhibit a discouraging prognosis characterized by a 5-year relative survival rate of about 68% in developing countries [1,2]. The WHO reports indicating that there are approximately 900,000 annual cases of HNCs, resulting in more than 400,000 deaths [3]. Particularly disheartening is the fact that developing countries experience even lower survival rates in this context [4,5,6,7]. Furthermore, oral cancer, encompassing approximately half of HNC instances, predominantly manifests as oral squamous cell carcinoma (OSCC), accounting for roughly 90% of these cases [8,9].

OSCC occurs within the oral cavity, encompassing vital areas such as the lips, gums, inner linings of the cheeks and lips, the front two-thirds of the tongue, the floor beneath the tongue, and the roof of the mouth [10]. Despite significant strides made in diagnostic techniques and the availability of various treatment approaches, the worldwide 5-year survival rate for OSCC continues to linger below 50%, underscoring the persistent challenges in effectively combatting this disease [11].

OSCC is characterized by an abundance of immune cells infiltrating the tumor, making it a tumor with high immunogenicity [12]. Within the tumor microenvironment (TME), which is a complex landscape consisting of the extracellular matrix (ECM), various stromal cells, and immune cells, there exists a dynamic interplay and coordination with the tumor cells. Examples of these interacting cells include tumor-associated macrophages (TAMs), regulatory T cells (Tregs), cancer-associated fibroblasts (CAFs), and endothelial cells [13]. Furthermore, the innate immune system contributes to the TME through macrophages, dendritic cells (DCs), neutrophils, myeloid-derived suppressor cells (MDSCs), natural killer cells (NKs), and innate lymphoid cells. The adaptive immune response is represented by T cells and B cells [14]. The communication and interaction among these cells, along with the ECM and tumor cells, play a significant role in driving tumor progression [15,16].

The utilization of three-dimensional (3D) models provides a novel avenue for investigating cancer immunology in preclinical studies. These models offer a more realistic and physiologically relevant environment compared to traditional two-dimensional (2D) models. Unlike murine models, 3D models are cost-effective, amenable to high-throughput research requirements, and can incorporate human cancers and immune components. The application of 3D models has significantly advanced various areas of cancer immunology research [17,18]. Among the commonly employed 3D models are spheroids, organoids, and microfluidic chips.

This paper aims to discuss the functions of immune cells related to tumors with a focus on in vitro cell culture technology. The review also provides an overview of the application of 3D models involved in oral microbial pathogenesis, drug discovery, and cell–cell interaction in carcinogenesis. Finally, the potential application of 3D models in the study of TAM functions in oral cancer is discussed.

## 2. Tumor Immune Microenvironment of Oral Cancer

The tumor immune microenvironment (TIME) of oral cancer is a complicated system composed of immune cells, cytokines, and chemokines that interact with tumor cells to promote or prevent tumor growth [19,20]. It is proven that the immunological microenvironment is vital in developing oral cancer and potentially serves as a target for cancer therapy [21,22].

In most cases, pro-inflammatory cytokines (IFN-γ, IL-2, IL-1α, IL-1β, TNF-α, IL-17, and IL-8), anti-inflammatory cytokines (IL-4 and IL-10), and pro-/anti-inflammatory cytokines (IL-6 and TGF-β) are unbalanced in oral cancer, resulting in an immunosuppressive environment. This immunosuppressive milieu permits cancer cells to avoid detection by the immune system [23,24]. A study showed how an unbalanced quantity of cytokines can act like a two-edged sword. In the study, IL-10, as an anti-inflammatory cytokine, exhibited an elevated expression, while TGF-β experienced a reduction. This situation ultimately led to the suppression of the immune system in the progressive stage of OSCC [25]. Therefore, gaining insights into the markers associated with oral cancer and comprehending the intricate dynamics between cancer cells, stromal cells, and the tumor microenvironment hold paramount significance in the realms of diagnosis and treatment. 

### 2.1. Interaction between the Immune System and Tumor Cells

The immune system has a dual role in tumor growth, either suppressing it or promoting it, known as immunoediting [26]. Initially, the immune system can eliminate tumor cells, a phase called immunosurveillance. During this stage, there are lively molecules and immune cells at work, capable of identifying and eliminating cancerous cells. Furthermore, perilous signals such as Type I interferons (IFNs) are discharged by dying tumor cells or injured tissues as the tumor advances. These signals rouse the immune cells and promote a flexible, tailored anti-tumor immune reaction. Certain molecules such as MHC class I chain-related protein A and B (MICA and MICB) and the histocompatibility 60 (H60), which are plentiful on the surface of tumor cells, bind to activation receptors found on immune cells, setting them into action. The distribution of cluster of differentiation (CD)4^+^ T and CD8^+^ T cells facilitates a harmonized and well-balanced activation of both the innate and adaptive immune responses [27]. 

If the defective cells are not completely removed, the equilibrium phase begins, where tumor cells remain dormant with the help of interleukin (IL)-12, T cells, and IFN-γ [28]. During this phase, the adaptive immune system becomes crucial, leading to two possible outcomes: regression and elimination of the tumor or its progression [29,30]. For example, it was observed that after oral cancer cells in the dormancy stage were exposed to irradiation, M2 TAMs actively induce neovasculogenesis, fostering the resurgence of oral cancer [31]. The escape phase, extensively studied in cancer immunoediting, often occurs due to T-cell exhaustion [32]. This exhaustion involves intrinsic mechanisms of T cells, such as the Programmed death-1 (PD-1)/Programmed Cell Death Ligand 1 (PD-L1) pathway and immunoregulatory receptors such as cytotoxic T-lymphocyte antigen 4(CTLA-4), T-cell immunoglobulin and mucin-domain containing-3 (TIM-3), and lymphocyte activation gene-3 (LAG-3), as well as extrinsic pathways mediated by Tregs and MDSCs. These pathways secrete cytokines such as transforming growth factor beta (TGF-β) [33]. The interaction between hypoxia-inducible factor (HIF)-1α and immune cells contributes to the evasion of the immune system by tumors, including OSCC [34].

### 2.2. Immune and Non-Immune Markers

One strategy for early detection of oral cancer is the identification of immune and non-immune markers [35,36]. T cells, B cells, and NKs, among many other immune and non-immune cells, are used to diagnose oral cancer [37,38,39]. Non-immune markers used in diagnosis include changes in DNA, RNA, and protein expression levels. These markers can help identify individuals who are most prone to being affected by the condition, allowing for early identification and treatment [40]. Immune and non-immune biomarkers are listed in Table 1.

### 2.3. Stromal Cell and Extracellular Matrix (ECM) on Cancer Immunity

The intricate makeup of the tumor microenvironment and the interactions between the tumor and its surrounding stroma play a pivotal role in fueling tumor growth and facilitating its spread, resulting in unfavorable clinical outcomes in understanding and defining the disease, as it exerts significant influence over cancer cell invasion, migration, angiogenesis, resistance to drugs [84,85], maintenance of cancer stem cells [86], and evasion of immune surveillance [87,88].

Within the TME, the tumor stroma represents the noncancerous components and consists of an abundant ECM and various supportive cell types [89]. These include CAFs, endothelial cells, pericytes, and immune cells such as lymphocytes, neutrophils, DCs, monocytes, and TAMs, which are the most prevalent cell types. Additionally, there are less common factors such as MDSCs and mesenchymal stromal cells (MSCs) [84,90], as well as platelets [87,91]. These stromal cells actively engage in intricate interactions with tumor cells, with one another, and with the ECM. They achieve this by releasing chemokines, growth factors (GFs), enzymes, extracellular vesicles, and miRNAs that regulate the expression of genes and proteins, thereby influencing metabolic pathways associated with cancer [92]. Consequently, different cell types can either promote or suppress tumor growth depending on the specific cellular context [93]. Current in vitro culture models come with numerous limitations, including a lack of the dynamic interplay between cells and their surrounding environment, and alterations in cell shape, orientation, and proliferation. These disadvantages prompted the development of alternative models that better simulate in vivo conditions. Among these approaches, 3D culture appeared to be a promising method to bridge the gap and led to a significant emphasis on developing accurate models to study and simulate TME interactions both in laboratory settings and in living organisms [94]. Table 2 shows the role and function of stromal cells in oral cancer.

## 3. In Vitro Models in Oral Cancer

**Figure 1:**
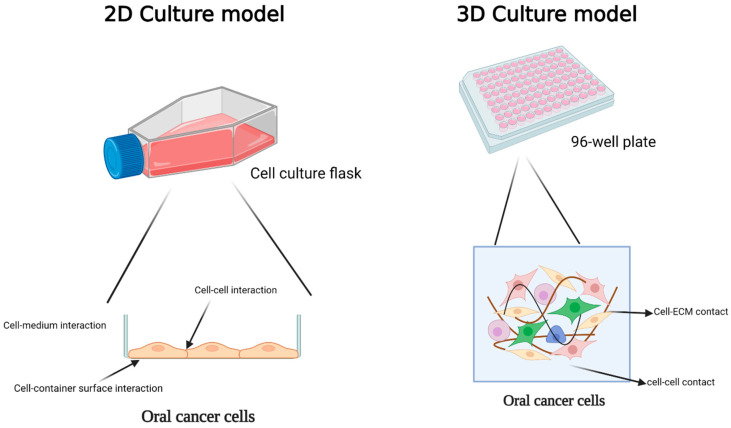
Comparison of 2D and 3D models for oral cancer studies and exploring cellular interactions in these conditions. In a 2D culture, cells establish connections with neighboring cells, the container’s surface, and the surrounding environment on one side. In 3D culture, the cell maintains efficient communication with neighboring cells and the extracellular matrix. Created with BioRender.com.

At present, the most common evaluation platform for drug development in oral cancer is conventional 2D in vitro models to their low cost, high reproducibility, and potential co-culture capability [134]. For example, cell co-culture in a 2D model helped in gaining a deeper comprehension of how CAFs interact with cancer cells within the microenvironment, revealing the potential for CAFs to regulate cancer cells as a means of therapeutic mediator [135]. However, 2D in vitro models are unable to mimic the physical geometry of the tumor, avoid the cross-contamination of culture media in multicellular models, and mimic the oxygen deprivation and irregular irrigation of the hypoxia region, which are key factors in the evaluation of tumor progression, chemoresistance, and treatment response [136,137]. Advanced in vitro systems, including spheroids, 3D scaffolds, and microfluidic devices, have thus been developed to overcome these barriers [138]. Although the application of these culture platforms to model the oral cancer microenvironment and its drug discovery is still in its infancy, recent research on oral cancer has used 3D in vitro models to advance the growing need for these systems for clinical translation, which is categorized into two forms, scaffold-free and scaffold-based strategies (Figure 1, Table 3).

### 3.1. Two-Dimensional Models

In the realm of in vitro cancer research, the traditional way of cultivating cells in a flat, 2D environment using culture flasks or Petri dishes has long been favored due to its cost-effectiveness, simplicity, and ability to yield consistent results. The application of 2D cell culture models is accepted as a means to evaluate how cells respond to potential drugs. While the acceptance and uses of 2D cell culture have undoubtedly contributed to the advancement of comprehending the mechanisms of drug action, it is not without its constraints [139,140]. Therefore, this straightforward approach falls short of capturing the intricate, diverse, and ever-changing nature of the TME due to a range of limitations. These drawbacks encompass insufficient cell differentiation, unrealistic cell growth patterns, diminished drug resistance, and inaccurate reactions to mechanical or chemical signals [141]. Consequently, employing this 2D culture technique creates notable discrepancies when compared to the dynamic conditions present in living organisms, encompassing factors such as tissue development, specialized cell functions, cell division, cellular migration, gene and protein expression, signal transduction, and responsiveness to stimuli [142,143].

Relying solely on the 2D pre-clinical model is far from ideal and can generate misleading research results, primarily because cells exhibit distinct behaviors when cultured in a 2D setting compared to when they are organized in a 3D structure [144]. The earliest indication of this phenomenon dates back to 1985 when Miller and colleagues made a noteworthy discovery: tumor cells cultivated as multicellular spheroids within a collagen gel displayed heightened resistance to drugs compared to cells grown as a single layer [145]. Recent studies exploring the response to chemotherapy and radiation treatment in both 2D and 3D spheroids have yielded similar findings. These investigations revealed that oral cancer cells cultured in 3D tumor spheroids exhibited enhanced resistance to radiation and greater viability, even when exposed to higher doses of cisplatin [146,147,148].

### 3.2. Three-Dimensional Models

**Figure 2:**
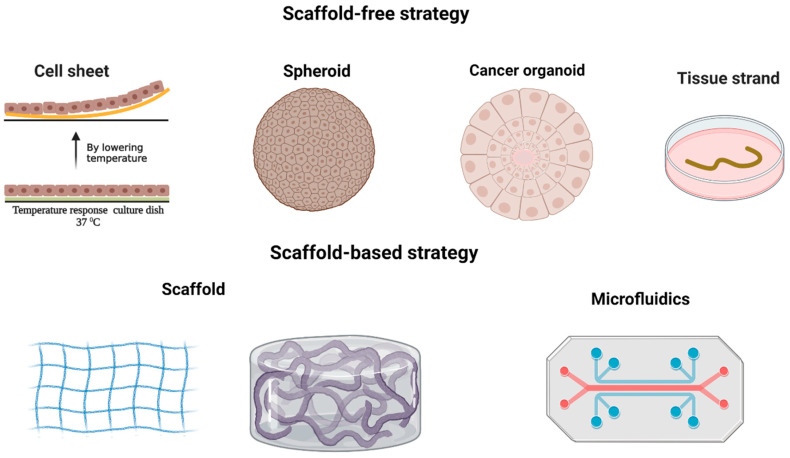
In cancer research, two distinct approaches are employed for 3D modeling. The first is the scaffold-free strategy, which encompasses cell sheet, spheroid, cancer organoid, and tissue strand techniques. The second is the scaffold-based strategy, involving the use of scaffolds and microfluidics. Created with BioRender.com.

In a bid to overcome the constraints inherent in flat cell cultures, scientists have ventured into the realm of creating advanced in vitro cancer models that embody 3D structures. It has been scientifically established that the essential functions of the ECM and its significance as a vital biomaterial in tissue engineering cannot be understated. When it comes to obtaining the ECM for tissue engineering, two distinct strategies come into play: the employment of scaffolds or the scaffold-free approach. Each approach brings forth its own set of advantages and disadvantages; thus, the purpose of the research is decisive in choosing a type of strategy. The scaffold-based approach entails the utilization of an external material as a substitute for the natural ECM. This method grants the ability to manipulate the mechanical and chemical aspects of the cell’s ECM, while concurrently providing enhanced structural integrity for the regeneration of larger tissues [149]. Conversely, the scaffold-free approach facilitates tissue formation by compelling cells to aggregate into small spheres, aptly named “spheroids”, which possess the capacity to develop an inherent ECM. This approach offers several benefits, including a high initial cell density, swift formation, and the creation of a self-organized tissue-like structure [150] (Figure 2). 

#### 3.2.1. Scaffold-Free Strategy

The scaffold-free method takes a unique “bottom-up” approach to constructing tissues. Instead of using scaffolds, it relies on small building blocks in three forms: spheroid, cell sheet, or tissue strands. These building blocks naturally combine and form larger structures. Unlike the scaffold-based technique, the scaffold-free method does not heavily rely on cell growth and movement, making the tissue construction process faster. One significant advantage of this approach is its capability to create complex tissue and organ structures by using diverse building blocks made up of different types of cells [150].

In a study, a cell sheet was made up of patient-specific epithelial and sub-epithelial cells to create a co-culture system for head and neck squamous cell carcinoma (HNSCC) patient-derived explants. The findings revealed that this co-culture system significantly enhanced the survival and longevity of the explants compared to other non-matrix models [151]. Moreover, the system also demonstrated a decrease in viable cancer cells following standard-of-care treatments. However, despite the potential of cell sheet cultural techniques, they are not easily accessible, challenging to establish, and expensive, which limits their widespread adoption [152]. In addition, there is no original study about tissue strand technology and oral cancer. Thus, most studies in oral cancer using the scaffold-free method focus on spheroids and their advanced form, the organoid, which is addressed in the following sections.

##### Spheroid

Tumor cells, when cultivated as spheroids, take on a 3D structure that emphasizes cell-to-cell connections rather than interactions with the culture substrate. These spheroids, unlike conventional 2D cell cultures, provide numerous benefits. They foster enhanced cell-to-cell communication and facilitate improved diffusion of vital substances and molecules. Multicellular tumor spheroids bear a striking resemblance to avascular tumor masses found in large solid tumors. They exhibit comparable characteristics in terms of appearance, growth dynamics, cell division, drug permeability, nutrient gradients, oxygen levels, and the formation of a hypoxic core [153].

As in the case of in vivo tumors, deep within the spheroid, cells encounter limited diffusion of nutrients, causing the development of a necrotic core. Additionally, the accumulation of metabolic waste and decreased nutrient availability leads to a notable decrease in the microenvironment’s pH. This pH gradient has the potential to affect drug release and influence the activity of genes related to multiple drug resistance, such as Multiple drug resistance (MDR). Consequently, therapeutics targeting hypoxic regions may face reduced uptake [154]. On the other hand, cells residing in the outer boundary of the spheroid (around 100–300 μm from the surface) enjoy better access to oxygen and nutrients, leading to heightened rates of proliferation [155].

Numerous investigations have showcased the potential of oral cancer spheroids as a valuable tool for replicating different aspects of tumor tissue, particularly its growth state and hypoxic conditions [156,157,158]. However, the utilization of this system is limited due to the spheroids’ constrained ability to self-renew and differentiate. Furthermore, spheroids can only mimic small-scale microenvironments since controlling their growth becomes challenging during culturing, resulting in central necrosis if they exceed a certain size [159]. Moreover, spheroids lack the intricate complexity found in actual tumor tissues, primarily because they are often composed of a single cell line and have minimal ECM deposition [153]. In response to these limitations, researchers have devised spheroids that incorporate diverse stromal components, such as CAFs, alongside epithelial HNSCC cancer cells. The aim is to establish a more representative tumor model that accounts for the crucial role of stromal cells in tumorigenesis [160].

##### Organoid

An organoid represents a more advanced version of a cellular spheroid that remarkably imitates the physicochemical conditions of a specific tissue. In contrast to spheroids, organoids possess a more organized cellular architecture when grown on specific matrices, resulting in a structured arrangement of cells [161]. Organoids can display various biological characteristics akin to living tissues, such as tissue organization, regenerative capacity, drug responses, and the retention of tumor heterogeneity [162]. Notably, Driehuis et al. [163] successfully generated human mucosal organoids from 31 patient-derived HNSCC samples originating from diverse head and neck regions. Through immunohistochemical and genetic analyses, it was revealed that these organoids preserved the histological traits and molecular abnormalities observed in the original tumor specimens. Functionally, the organoids exhibited distinct sensitivity to a range of drugs, including cisplatin, carboplatin, cetuximab, and radiotherapy, highlighting their potential for drug screening in HNSCC. Furthermore, when transplanted beneath the skin of mice, the organoids displayed atypical features, tripolar mitotic figures, nuclear pleomorphism, and invasion into muscle tissue, showcasing their tumorigenic abilities in vivo. These findings indicate that HNSCC organoids faithfully replicate the genetic, histological, and functional aspects of HNSCC and hold promise as a platform for personalized cancer therapy. 

Nevertheless, despite the benefits of organoids, there remain various limitations that necessitate attention. Tumor organoids usually recreate tumors originating from a single organ and fail to capture the intricate nature of cancer metastasis that involves multiple organs. Additionally, organoid cultures do not faithfully reproduce the spatial arrangement of cellular and non-cellular constituents within the tumor microenvironment [162]. Consequently, there are discrepancies in organoid complexity, size, morphology, and 3D structure, posing challenges in the standardization of organoid culture protocols [164].

#### 3.2.2. Scaffold-Based Strategy

Scaffolds crafted from natural substances such as collagen, Matrigel, and silk, as well as synthetic materials including polyethylene glycol (PEG) and poly(lactic-co-glycolic acid) (PLGA), or a combination thereof, offer robust support for cell development and can emulate numerous mechanical and biochemical characteristics of the original ECM [165]. The approach of utilizing scaffolds has been extensively employed to engineer diverse oral tissues, facilitating the exploration of underlying cancer mechanisms. For instance, scientists have ingeniously created human-tissue-engineered oral mucosal models (TEOMMs) by seeding keratinocytes onto matrices populated with fibroblasts and culturing them at the interface between air and liquid [166]. TEOMMs have enabled researchers to capture distinct stages of OSCC, encompassing oral dysplasia, early invasive OSCC, and the neoplastic transformation associated with stromal fibroblasts [167,168,169]. As an illustration, Sawant et al. [170] developed a TEOMM representing normal, dysplastic, and malignant tongue tissues to investigate the neoplastic progression throughout various stages of tongue tumorigenesis. Histomorphometry, immunohistochemistry, and electron microscopy analyses performed on the three types of models demonstrated comparable stratified growth, cell proliferation, and differentiation between co-cultures and their respective native tissues. These findings suggest that crucial steps in the development of oral tumors can be replicated in vitro using scaffold-based engineered tissues.

##### Limitation and Improvement of 3D Model

However, it is crucial to acknowledge that the distinctions between traditional 3D models and the authentic TME go beyond mere dimensional variations. The development of cancer involves a multifaceted process influenced by numerous cues that synergistically manifest cancer hallmarks. Consequently, to capture in vivo scenarios with greater fidelity, it is imperative to devise biomimetic in vitro alternatives. In general, the conventional in vitro 3D models face notable limitations, including the constrained potential for vascularization and the absence of well-arranged spatial distribution of tumor cells and ECM compositions. These factors are pivotal aspects of the native TME. The 3D in vitro models incorporate co-culture of cancer cells with several types of stromal cells to mimic tumor characteristics in vivo. In vitro, 3D culture revealed more aggressive behaviors and resistance to anticancer medicines than 2D culture, underlining the potential use of chemotherapy screening [134]. Advanced cancer screening models have the potential to reduce drug development costs by reducing the number of animals required for clinical trials [171].

To tackle this obstacle, the utilization of microfluidic platforms presents a promising solution to mimic complex and dynamic tumor microenvironments. These platforms, intricately crafted at a microscopic scale, consist of interconnected chambers, membranes, and grooves that facilitate the controlled manipulation of small fluid volumes. They have found extensive applications in the field of in vitro modeling, including the development of organ-on-a-chip models [172,173,174,175] and point-of-care systems [176]. Within the realm of cancer research, microfluidic platforms are predominantly constructed using advanced techniques such as lithography and surface micromachining, employing materials such as polydimethylsiloxane, silicon, glass, polycarbonate, and polymethylmethacrylate [172,174,177,178].

## 4. Application of 3D Model in Oral Cancer

### 4.1. Oral Microbiota Study

OSCCs, which refer to malignant growths in the epithelial lining of the lip and oral cavity, contribute significantly to global mortality and morbidity [1]. The primary culprits behind OSCC are believed to be tobacco and alcohol consumption. However, recent research suggests that factors such as inflammation and the oral microbiome also play a role in developing oral cancer. The presence of bacterial dysbiosis in surface lesion samples of OSCC is characterized by significant alterations in bacterial composition and bacterial gene functions compared to control samples. Specifically, a notable enrichment of periodontitis-associated taxa within the OSCC samples was observed, including *Fusobacterium*, *Dialister*, *Peptostreptococcus*, *Filifactor*, *Peptococcus*, *Catonella*, and *Parvimonas* [179]. Several inflammatory mediators, salivary proteins, and oral microbiota are shared between inflammatory conditions of the oral cavity (IL-1, IL-6, IL-8, TNF-α), such as periodontitis, and oral cancer [180,181].

To imitate the interactions between a host and microorganisms in the mouth, an ideal host model should possess the ability to support a viable microbial community. It should facilitate the exchange of metabolites and nutrients with the microbes, interpret microbial signals, respond accordingly, and eventually achieve a state of balance within a specific timeframe [182].

Due to their increased physiological relevance, 3D host models are becoming increasingly valuable for both fundamental research and clinical applications. In the current field of oral (dental) research, animal models and 2D cell models continue to be heavily relied upon for mechanistic studies and pre-clinical testing of oral hygiene products and medications. These studies encompass various areas, such as the treatment of salivary secretory disorders, oral wound healing, and oral cancers [183,184,185]. Since hard tissues contain relatively few organic components, studies on corresponding host–microbe interactions have predominantly focused on biofilms formed on inorganic surfaces, extensively exploring their metabolism, composition, and structures [186,187,188].

**Figure 3:**
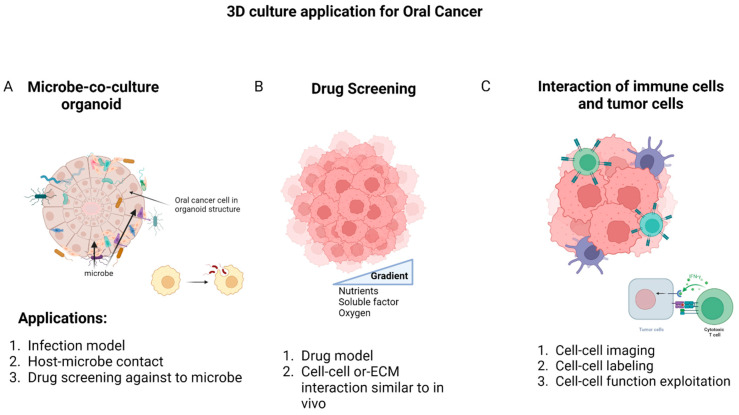
The most common application of 3D culture in oral cancer is related to examining the microbial population in oral cancer, drug screening, and the interaction between immune cells and cancer cells. (**A**) The first application can be a suitable model for investigating infection; it facilitates the study of the relationship between cells and microbes, and is ideal for the screening of effective drugs for infection control in oral cancer. (**B**) The second approach offers a solution to explore drug resistance mechanisms and examine crucial cellular interactions more effectively. (**C**) Finally, the investigation of interactions between oral cancer cells and immune cells involves labeling and imaging cell contacts to identify cell functions. Created with BioRender.com.

The earliest organotypic models of the host involved using tissue explants embedded in or cultured on various types of 3D gels to create a structure resembling an organ [189]. In the case of oral 3D models, the tissue is obtained by directly removing a section of oral mucosa from a human or animal [190]. However, due to ethical concerns and the limited availability of human oral mucosa, along with significant variations between donors [191], researchers began developing in vitro reconstructed organotypic models as a scalable and reproducible alternative. These models were designed to replicate important biological characteristics of the oral epithelium or oral mucosa (gingiva), including the 3D tissue architecture and the communication between different types of host cells and ECM. Organotypic models of oral cancer have proven their worth in the discovery of novel drug targets, evaluating the effectiveness of drug delivery systems, and confirming the biosafety and compatibility of graft materials for oral-maxillofacial surgery. Extensive reviews of these models can be found elsewhere [192,193,194] (Figure 3A). However, despite the significant impact of the oral microbiota on host physiology and pathology, it has not been widely incorporated into these models. In the studies that have begun to address this, researchers have employed various representative microorganisms to imitate host–microbe interactions in different oral niches.

Various types of cells were used as host cell sources in 3D oral host–microbe interaction models, including cancer cell lines [195,196], immortalized cell lines [197,198], and primary cells [186,199]. Studies utilizing these different cell sources showed similar outcomes when exposed to specific microbes, such as *Candida albicans*, in terms of tissue histology, Lactate dehydrogenase (LDH release), Human beta-defensin-2 (hBD2) expression, and secretions of TNF-α, IL-1β, and CXCL-8 [200].

When exposed to commensal microbial species, reconstructed human mucosal (RHM) models using either primary gingival cells or immortalized telomerase reverse transcriptase (TERT) cell lines exhibited similar responses. They remained viable and showed protective responses, such as activation of toll-like receptor (TLR) signaling pathways, increased expression of antimicrobial peptides (Elafin, hBD-2, hBD-3), and release of cytokines (IL-6, CXCL8, CXCL1, and C-C Motif Chemokine Ligand (CCL) 20) (Shang et al., 2018, 2019, 2020). Following exposure to a pathogenic biofilm composed of five species, two RHM models constructed with different keratinocyte cell lines demonstrated comparable levels of tissue damage, which were lower than those observed in a commercial reconstructed human epidermis (RHE) model after the same biofilm exposure [195]. Another study suggested a slightly higher invasion of *Porphyromonas gingivalis* occurred in 3D models using the H357 cell line (Human oral squamous cell carcinoma) compared to primary keratinocytes, but the difference was not significant [201].

### 4.2. Drug Discovery

Currently, the most commonly used method to evaluate drug development for HNCs is the conventional 2D in vitro model. These models are favored due to their low cost, high reproducibility, and potential for co-culture capabilities [134]. However, there are certain limitations with 2D models that need to be addressed. Firstly, they are unable to replicate the physical structure of tumors. Secondly, they cannot prevent the mixing of culture media in multicellular models, which can lead to cross-contamination. Lastly, they fail to simulate the oxygen deprivation and irregular blood flow in the hypoxia region, which are crucial factors in assessing tumor progression, chemoresistance, and treatment response [136,137]. To overcome these challenges, advanced in vitro systems such as spheroids, 3D scaffolds, and microfluidic devices have been developed [138]. Although the use of these culture platforms to model the HNCs and oral cancer microenvironment, and facilitate drug discovery, is still in its early stages, recent research in HNCs has shown promising results using 3D in vitro models. These advancements are essential to meet the increasing demand for improved systems for clinical translation (Table 4, Figure 3B).

### 4.3. Cell–Cell Interactions

Understanding the progression of cancer heavily relies on comprehending the intricate microenvironment in which malignant tumor cells thrive [219]. The physical and biochemical attributes of this environment play a pivotal role in regulating various aspects of cancer, including cell differentiation, proliferation, invasion, and metastasis [220]. Thus, it is imperative to gain insights into the interactions and communication between cancer cells and their surrounding tissue, referred to as the tumor stroma, as this interplay profoundly influences the advancement of the disease. In order to mimic the conditions of TME, 3D in vitro models have become widely employed [221,222] (Figure 3C).

The use of 3D organoid models in co-culture systems has emerged as a prominent area of research, particularly in the evaluation of anticancer properties. These models aim to mimic the complex interactions occurring within the tumor microenvironment [223]. Likewise, the exchange of signals between stromal cells and cancer cells through paracrine signaling has been recognized to promote both cell proliferation and the development of drug resistance. In Table 5, various model systems employed in studies related to HNCs are presented. These models offer the advantage of customization according to the specific parameters being investigated, surpassing traditional techniques. For example, to understand the role of monocytes, they can be cultured alongside HNC cells [224,225,226,227]. Similarly, fibroblasts and peripheral blood mononuclear cells (PBMCs) can be co-cultured with HNC cells for epidermal growth factor receptor (EGFR)-related investigations [228] and antibody testing [229], respectively.

Several previous studies have provided evidence regarding the involvement of TAMs and Human Dermal Fibroblasts (HDFs) in cancer stemness and invasion, respectively [230,231]. The response to drugs targeting EGFR has been investigated with CAFs, revealing variations [214]. Various organoid models of HNCs have been established to investigate signaling pathways, such as Extracellular signal-regulated kinase 1/2 (ERK1/2) and Nanog [232], as well as their interactions with Human herpes simplex-1 (HSV1) and papillomavirus type 16 (HPV16) [163], the invasiveness of cancer [233], drug screening [234], and other defining characteristics [235]. An innovative microfluidics platform called the Hydrodynamic Shuttling Chip (HSC) has been developed to separate and co-culture single-cell SCC with lymphatic endothelial cells, enabling the observation of cell motility and intercellular communication [236].

**Table 5:** Interactions of oral cancer cells and immune cells.

3D Model	Aim	Result	References
Co-culture of monocytes with spheroids originating Malignant/benign HNC	The connection between the response of this cytokine co-culture and the prediction of outcomes.	The secretion of IL-6 during in vitro co-culture with monocytes and BF ^1^-spheroids serves as a prognostic indicator for recurrence and overall prognosis, whereas co-culture with monocytes and MF ^2^-spheroids predicts the likelihood of recurrence.	[227]
Co-culture of HNC cell line with fibroblasts in spheroid form	Generation of a spheroid model of EGFR-expressing HNC.	The upregulation of chemokine expression by anti-EGFR mAb ^3^ promotes the infiltration of leukocytes into tumor spheroids. This unique mechanism of action of anti-EGFR mAb could potentially enhance the anti-tumor effects of the antibody in living organisms.	[228]
Spheroid form of HNC cell line culture with leukocytes from PBMC ^4^	The evaluation of utilizing a 3D tumor cell culture model, specifically spheroids, as a suitable representation of micro-metastases.	The utilization of the spheroid model demonstrates the manifestation of pathophysiological traits, intricacy, and heterogeneity of tumor tissue observed in vivo, which significantly impacts the effectiveness of therapeutic interventions.	[229]
Co culture of HNC spheroids with TAMs	The signaling of CD44, influenced by TAMs, has the potential to facilitate stemness through the PI3K-4EBP1-SOX2 pathway. This effect may occur by regulating the availability of HA ^5^, which is the primary ligand for CD44.	The results establish a mechanistic connection between CD44 in tumor cells, TAMs, and the properties of CSCs ^6^ at the interface between tumor and stroma. This connection highlights a crucial area for targeting and discovering drugs.	[231]
Co-culture of HNC cell line with HDFs	The understanding of how cancer cells, fibroblasts, and the surrounding collagen matrices interact and promote cancer cell invasion in different environments with varying concentrations of collagen.	The presence of HDFs played a crucial role in facilitating the invasion of HNC cells into the surrounding extracellular matrix characterized by high collagen concentration, elevated storage modulus, and narrow pore sizes.	[230]
Co-culture of HNC cell line with CAFS	Assessing the impact of CAFs on the treatment response and migratory behavior of HNC.	The presence of CAFs resulted in enhanced cell proliferation within the tumor spheroids, which was accompanied by elevated EGFR expression. Notably, spheroids exhibiting heightened EGFR expression displayed an augmented response to cetuximab treatment.	[210]
HNC spheroids	Role of ERK1/2-Nanog pathway in tumorigenesis in HNC.	HNSCCs sustain a population of CSCs by utilizing the ERK1/2 signaling pathway and Nanog.	[232]
Oral mucosal Organoids and HNC patient-derived tumoroids	In vitro 3D model for HNC.	Drug screening for both existing and experimental therapeutic treatments for HNC.	[163]
HNC spheroids	The correlation of CD44 and HIF-1α expression.	By focusing on HIF-1α, the impact of NOTCH1-induced stemness, which controls the reaction to chemotherapy or radiotherapy as well as the malignancy in CD44^+^ HNSCCs, was reduced. Targeting the signaling of HIF-1α/NOTCH1 could potentially serve as a therapeutic approach for the treatment of HNSCC.	[237]
Co-culture of OSCC cell line with CAFS	Role of stromal NNMT ^7^ in TME.	The harmful cancer-promoting effects caused by stromal NNMT were reduced when fibroblasts were treated with inhibitors targeting collagen production, such as losartan, tranilast, and halofuginone.	[238]

^1^ benign, ^2^ malignant, ^3^ monoclonal antibody, ^4^ peripheral blood mononuclear cells, ^5^ hyaluronic acid, ^6^ cancer stem cells, ^7^ Nicotinamide *N*-methyltransferase.

## 5. Potential Application of 3D Model in Studying TAM Functions in Oral Cancer

Within the TME, immune cell components, notably TAMs [239,240], play a dominant role in suppressing the adaptive immune system through various pathways [241,242].

TAMs employ multiple strategies to hinder the activity of immune cells. They express receptor molecules for immune checkpoint proteins, including PD-1 and CTLA-4, while also releasing cytokines and chemokines that trigger Treg-mediated suppression pathways [243]. This interplay between TAMs and other immune elements is bidirectional. 

It is crucial to utilize 3D in vitro tumor models to investigate the interaction between tumor cells and their microenvironment in a biologically relevant manner. Organotypic co-cultures have been previously employed to examine the malignant growth of human SCC cell lines in vitro, where the cell lines are cultivated on a collagen-I gel containing fibroblasts to mimic the stromal environment [244]. Recognizing the significant role played by macrophages in tumor progression, this model was further developed by incorporating macrophages into the collagen gel of these organotypic tumor co-cultures. This approach was established as both a murine and a human system for studying skin SCCs. The impact of macrophages on tumor progression relies on their polarization state. It was demonstrated that in organotypic co-cultures, the polarization of macrophages can be directed towards either an M1 or an M2 phenotype by introducing recombinant IFN-γ and lipopolysaccharide (LPS) or IL-4, respectively, into the growth medium. Stimulation of macrophages with IL-4 in these cultures led to an intensified invasion of tumor cells, as evidenced by basement membrane degradation, increased collagenolytic activity, and elevated levels of MMP-2 and MMP-9. Interestingly, prolonged co-culture with tumor cells for three weeks resulted in spontaneous M2 polarization of macrophages without the need for IL-4 treatment. Thus, the integration of macrophages into organotypic co-cultures of murine or human skin SCCs has proven to be successful, providing a valuable model for investigating the activation of macrophages towards a phenotype that supports tumor growth [245].

The influence of different macrophage phenotypes on cancer cells was examined, revealing that activated macrophages of the M2 phenotype had a substantial impact on promoting the proliferation, migration, and invasion abilities of cancer cells. To mimic the tumor microenvironment, co-cultures of M2 macrophages and cancer cells were established within agarose hydrogels. It was confirmed that the expression of CXCL2, a chemokine, was notably higher in the co-culture system. Functional analysis data indicated that the addition of recombinant human CXCL2 significantly enhanced the migration and invasion abilities of cancer cells while impairing their adhesion ability. Notably, the effects of CXCL2 on cancer cells were effectively diminished by neutralizing CXCL2 or blocking its receptor, CXCR2. This observation suggests that CXCL2 may play a critical role in facilitating metastasis [246].

TAMs exhibit a mixed phenotype combining characteristics of both M1 and M2 macrophages, and their distribution within the tumor microenvironment is believed to be dynamic and evolving throughout tumor progression [247,248,249]. However, the understanding of how TAMs specifically influence the tumor microenvironment is limited by the absence of suitable 3D in vitro models that can capture the intricate dynamics between cells with high spatial and temporal resolution. To address this, a novel microphysiological “tumor-on-a-chip” (TOC) device was employed to investigate the impact of distinct subsets of macrophages on tumor behavior. The TOC device features three interconnected chambers, with tumor cells loaded in both the upper and lower chambers, while the central chamber contains a living microvascular network that can be perfused. When human pancreatic or colorectal cancer cells were introduced alongside M1-polarized macrophages, a significant inhibition of tumor growth and tumor-induced angiogenesis was observed. Detailed protein analysis and neutralization studies using specific antibodies confirmed that these effects were mediated through the production of C-X-C motif chemokines (CXCL9), CXCL10, and CXCL11 by the M1 macrophages. In contrast, M2 macrophages facilitated increased migration of tumor cells into the vascularized chamber, without inhibiting tumor growth or angiogenesis. This innovative TOC model provides valuable insights into the complex interactions between TAMs and tumor cells, shedding light on the contrasting roles played by M1 and M2 macrophages in the tumor microenvironment [250].

Macrophages, commonly found within the TME, actively collaborate with cancer cells during the invasion process [251,252], coinciding with the presence of elevated interstitial flow (IF) levels [253,254]. Although studies have extensively explored the biochemical mechanisms driving pro-tumor macrophage functions, IF within the TME has often been neglected [239,255]. The concept of a 3D microfluidic model revolves around understanding the dynamics of fluid movement within micro-sized channels and the advanced techniques involved in fabricating miniature devices comprising chambers and tunnels that serve as pathways or confinement spaces for fluid flow [256]. Therefore, a novel 3D microfluidic model was devised to investigate how IF impacts macrophage migration and its potential contribution to cancer invasion when combined with tumor cells [257]. Interestingly, the presence of either tumor cells or IF individually heightened macrophage migration in terms of directionality and speed. However, when tumor cells and IF were simultaneously present, no additional effect on macrophage migration directionality and speed was observed [257].

## 6. Conclusions and Future Perspective

Over the past decade, remarkable progress has been made in unraveling the mysteries of the immune system’s fight against cancer. These advancements have spanned a wide range, from fundamental scientific research to practical clinical studies. As our understanding of the underlying biology becomes more intricate, it is essential to employ models that can capture this complexity. Three-dimensional culture systems have emerged as invaluable tools that offer both complexity and interpretability.

Geometry, multicellularity, and continuous irrigation are important characteristics for generating oral-cancer-specific in vitro models for drug screening and development. Organotypic multicellular spheroid and organoid cultures are useful for simulating cancer-specific TME by reflecting required shape and cell–cell/–ECM interactions found in tumor tissues. Organotypic models can be integrated with microfluidic devices to assess cell-to-cell communication and obstacles to mass transport of oxygen, nutrients, and medicinal therapies. The use of a tumor-on-a-chip technology is intended to reduce the requirement for animal models and the likelihood of clinical trial failures in translational research.

The application of 3D culture systems has revolutionized cancer research, shedding light on various aspects such as the distribution of immunotherapy, the infiltration of immune cells, and the suppression of the immune system caused by cancer. These systems have provided new insights and deepened our understanding in these areas and beyond. However, there are still untapped areas in the field of cancer immunology where 3D culture systems have yet to make their mark.

For instance, understanding the migration and function of Treg cells, unraveling the biology of myeloid-derived suppressor cells, and advancing vaccine development are promising avenues that could benefit from the application of 3D culture systems. As the cost of 3D technology decreases and accessibility improves, and as we explore the conceptual possibilities of these systems, it is highly likely that their popularity will continue to soar. In fact, they may even replace 2D culture methods altogether, marking a new era in cancer research and therapy.

Hence, 3D culture systems present an immense prospect for investigating cancer immunology; however, they do come with certain limitations. The heightened intricacy of these systems can pose challenges when it comes to reproducing results both within and between experiments. Additionally, compared to 2D culture systems, 3D culture systems are costlier and less readily accessible. Some microscopy techniques may struggle to capture images of 3D cultures due to their depth and limited transparency.

When it comes to utilizing 3D culture systems for studying cancer immunology, maintaining primary immune cells in culture for extended periods can be difficult, regardless of whether it is in a 2D or 3D environment. Moreover, the extraordinary complexity of the immune system, with its multiple steps and interactions between various cell types, may restrict the applicability of simple heterotypic or multicellular culture methods.

However, there is good news on the horizon. As technology continues to advance, the accessibility, versatility, and relevance of 3D models are expected to expand concurrently. This suggests that the limitations currently associated with 3D culture systems can be overcome with further developments, opening up even more possibilities for exploring cancer immunology in the future.

## Figures and Tables

**Table 1:** Immune and non-immune markers in oral cancer.

Type	Secretory Cell	Markers	References
Immune Cell	M1 TAMs	CD11c, CD80, HLA-DR ^1^	[41,42,43]
	M2 TAMs	CD163, CD11b, CD206, MRC1 ^2^	[43,44,45,46]
	DC	S100, CD1a, CD83, CD207, CD208, CD80, CD11c, CD86, HLA-DR CLEC9A ^3^	[47,48,49]
	NK cells	CD57	[50,51,52]
	pan T cell	CD3	[12,53,54]
	cytotoxic T cell	CD8	[12,55,56]
	T helper cell	CD4	[12,57,58]
	Pan B cell	CD19, CD20	[12,59,60]
	MDSCs	CD33, CD11b	[61,62,63]
	CAFs	α SMA ^4^	[64,65,66]
	Treg	FOXP3^+^ CD4^+^ T cells or CD4^+^ CD25^+^ CD127^low^	[39,67,68]
Non-immune cell	endothelial cells	CD34	[69,70,71]
	Salivary biomarkers	L-phenylalanine Sphinganine Phytosphingosine S-carboxymethyl-L-cysteine	[72,73,74]
	Genomic biomarkers	ITGA3 ^5^, ITGB4 expression	[75,76,77]
	Oral cancer cell	CCR7 ^6^	[78,79,80]
	Oral cancer cell	MYO1B ^7^	[81,82,83]

^1^ Human Leukocyte Antigen-DR isotype, ^2^ Mannose Receptor C-Type 1, ^3^ C-type lectin domain family 9 member A, ^4^ Alpha-Smooth Muscle Actin, ^5^ Integrin Subunit Alpha 3, ^6^ C-C chemokine receptor type 7, ^7^ Myosin IB.

**Table 2:** Mechanism and function of stromal cells in OSCC.

Stromal Cells	Mechanism	Function	References
CAFs	Production of numerous ECM proteins such as HAS2 ^1^ expression by CAFs	Tumor cell invasion by increasing ECM-degrading MMPs ^2^ and decreasing TIMPs ^3^	[95,96]
	high expression of α-SMA in CAFs	OSCC invasion into the bone by increasing expression of RANKL ^4^ and OPG ^5^	[97,98,99,100]
	High secretion of IL-1α by OSCC upregulated expression of secretory cytokines, including CCL7 ^6^, CXCL1 ^7,^ and IL-8	Tumor cell proliferation	[101,102,103]
	IGF-1 overexpression in CAF and activation of PI3K-AKT and Hedgehog signaling pathways	Tumor cell proliferation, migration invasion, tumorsphere formation angiogenesis.	[104,105,106]
	Overexpression of NOTCH-1 in CAFs	Increasing tumor volume angiogenesis in OSCC	[107,108,109]
	Overexpression of IL-6 in CAFs	Expression of VGEF in CAFs and OSCC angiogenesis in OSCC	[110,111,112]
	Multiple factors derived from CAFs, such as CXCL12 and MCP-1 attract macrophages to tumors and induce the M2 phenotype	A mediator for T-cell suppression	[113,114]
	IL-1α secreted from OSCC cells induces the chemokine CCL7 in co-cultured CAF	OSCC invasion and progression	[115,116]
TAMs	increased expression of arginase I, IL-10 and TGF-β	Suppressive effect on T cells and invasion and metastasis of OSCC	[117,118]
	PDL-1 and IL-10 production in TAMs	Immune escape of OSCC cells	[119,120]
	the secretion of EGFs ^8^ and the management of collagen production by TAMs	OSCC invasion and progression	[121,122]
	EMT ^9^ induced by TAMs decreased E-mucin and E-cadherin and increased-vimentin protein in OSCC cells	OSCC invasion and progression	[123,124,125]
	activated the Hh ^10^ signaling pathway by TAMs	Angiogenesis in OSCC	[126,127]
	Activation of TGF-β1/TβRII/Smad3 signaling pathway in TAMs	VEGF secretions in OSCC	[128,129]
	TAM number modulation by PFKFB3 ^11^	Angiogenesis in OSCC	[130,131]
DCs	activated the TNF-α/NF-κB/CXCR-4 pathway by pDCs ^12^	Oral cancer proliferation and invasion	[132,133]

^1^ hyaluronan synthetase 2, ^2^ matrix metalloproteinases, ^3^ tissue inhibitors of metalloproteinases, ^4^ receptor activator of NF-κB ligand, ^5^ tumor necrosis factor receptor superfamily member 11B, ^6^ chemokine ligand 7, ^7^ C-X-C motif chemokine 1, ^8^ epidermal growth factors, ^9^ Epithelial-mesenchymal transition, ^10^ Hedgehog, ^11^ 6-phosphofructo-2-kinase/fructose-2, 6-biphosphatase 3, ^12^ plasmacytoid dendritic cells.

**Table 3:** Comparison among 2D model, 3D model, and animal model.

	2D Model	3D Model	Animal Model
Modeling human development and disease	-	+	+
High costs, high personal, and work effort	-	-	+
High-throughput screening	+	+	-
Personalized medicine	-	+	-
Vascularization and immune system	-	-	+
Architecture	-	+	+

The signs indicate - and +, -: negative, and +: positive.

**Table 4:** 3D models for drug discovery in OSCC and HNCs.

3D Models	Drug	Application	Reference
Spheroid	Cetuximab Cisplatin	To assess the impact of both 2D and 3D culture techniques on gene expression related to cell junctions, cell adhesions, cell cycle, and metabolism. To verify the feasibility and practicality of this novel 3D culture approach for oral cancer research.	[147,202,203,204]
	Cisplatin Doxorubicin Methotrexate	To evaluate the differences in chemoresistance between 2D and 3D culture methods.	[205,206,207]
	Cisplatin 5-FU 2-Gy Radiation	To assess and compare the efficacy of 2D and 3D methods as platforms for chemotherapy and radiotherapy testing.	[208,209]
	Cetuximab	To develop a biologically significant in vitro model of HNSCC that accurately replicates the tumor environment by incorporating both tumor cells and CAFs in a 3D culture system.	[210]
Organotypic models	Cisplatin Docetaxel ± 5-FU	To evaluate and compare the suitability of 2D and 3D methods as platforms for assessing chemotherapy sensitivity.	[151,211,212]
	Cisplatin ± (carboplatin, cetuximab, radiotherapy)	To assess and compare the effectiveness of 2D and 3D methods as platforms for chemotherapy screening and regenerative purposes.	[163,213]
	Cetuximab mTOR inhibitor Canertinib Dactolisib PF-04691502 Apitolisib Omipalisib Refametinib binimetinib trametinib pimasertib trametinib	To evaluate and compare the efficacy of 2D and 3D methods as platforms for dual drug screening.	[214,215]
Microfluidic platforms	5-FU Cisplatin ± (Paclitaxel, Cetuximab, Carboplatin)	The use of a dynamic culture method as a platform for chemotherapy screening.	[175,216,217]
	IDO1 inhibitor ± (PDL1 antibody, Nivolumab)		[172,218]

## Data Availability

Data will be made available on request.
